# Belonging Exacerbates the Relations Between Racial Climate Stress and Physiological Dysregulation

**DOI:** 10.1007/s40615-023-01740-0

**Published:** 2023-09-07

**Authors:** Stacey N. Doan, Alicia S. Davis, Molly Lazarus, Akriti Poudel, Phil Tran, Natalie Clark, Thomas E. Fuller-Rowell

**Affiliations:** 1 Claremont McKenna College, Department of Psychological Science, Claremont, CA 91711 USA; 2 City of Hope National Medical Center, Department of Population Sciences, Duarte, CA USA; 3 Auburn University, College of Human Sciences, Human Development and Family Science Duarte, Auburn, CA USA

**Keywords:** allostatic load, racial climate stress, belonging, emerging adults

## Abstract

**Objective:**

Belonging is often considered a buffer against the physical and emotional consequences of discrimination and racial climate stress Youth Soc. 48(5):649–72, 2016. However, recent research suggests that feelings of belonging toward an institution can be detrimental when an individual feels discriminated against by the same institution to which one feels a sense of connection J Behav Med. 44(4):571–8, 2021. Therefore, the present study aimed to investigate the moderating role of institutional belonging in the relationship between racial climate stress and health, as indexed by allostatic load (AL), a multi-system indicator of physiological dysregulation.

**Methods:**

In a sample of Black and White college students (*N* = 150; White = 82; Black = 68), self-reported racial climate stress, institutional belonging, and various demographic variables were collected. An AL composite was also collected, comprised of six biological measures of the SAM system, HPA axis, cardiovascular system, and metabolic system. Multiple regression analyses were conducted to explore the relationships between these variables.

**Results:**

Results demonstrated no main effect of racial climate stress on AL but did show a significant interaction between racial climate stress and belonging, such that the positive relationship between racial climate stress and AL was significant only for those who also felt high levels of institutional belonging (*β*_*int*_ = .05, *p* = .006, 95% CI = 0.01 – 0.08).

**Conclusions:**

Feeling a sense of belonging may have negative physiological consequences for those who experience racial climate stress in a college setting.

Educational attainment has been associated with positive outcomes including health and well-being [3]. At the same time, research suggests that the social environments of colleges can be detrimental to students leading to increased stress and poor health behaviors [4]. Racial climate stress, in particular, can be detrimental for students from ethnic-minority backgrounds (e.g., [5]). The effects of campus climate are likely to depend on the individual’s sense of belonging, giving extant evidence for its primary role in affecting college student adjustment [6]. Although social belonging is often conceptualized as a buffer against the deleterious effects of discrimination (e.g., [7]), social identity theory would suggest that in certain contexts belonging can exacerbate negative relations between racial climate stress and health [2]. The present work investigates racial climate stress and allostatic load, a multi-system indicator of chronic physiological stress in a sample of Black and White college students and the moderating role of belonging.

## Perceived Racial Climate Stress

Racial climate refers to “behaviors, practices, and attitudes that together reflect the level of acceptance or rejection of racial diversity in a given institution” [5] and is common in the lives of minority college students [8]. While racial climate stress and discrimination are related, they are also distinct in that the former reflects a general perception of an overall climate, while assessment of discrimination generally focuses on individual, interpersonal experiences. To illustrate the distinction, racial climate stress can be perceived regardless of direct experiences of discrimination [5]. At the same time, discriminatory experiences can contribute to negative perceptions of racial climate (e.g., [9–11]). Individual differences in experiences of racial discrimination are also associated with negative perceptions of racial climate in both Black and White students, but the relationship is stronger for Black students [12].

Research has demonstrated the negative effects of perceived racial climate. Pieterse et al. [5] found that perceived negative racial climate significantly explained symptoms of post-traumatic stress disorder in Black students. Relatedly, perceived racial climate related stress is associated with worst mental health outcomes among minority college students [13]. White students who perceive negative climates can also exhibit detrimental educational outcomes. Cabrera et al. [14] investigated the outcomes of campus racial climate, such as social experiences and persistence, finding comparable perceptions of racial climate and discrimination in White and Black students. They also found that among students of both races, those who perceived discrimination had poorer persistence outcomes than those who did not.

The majority of work on racial climate stress in college has focused on mental health [5, 13] and few have considered its effects on objective markers of physical health. However, research on stress in general has found that repeated encounters with stress can lead to chronic overactivation of physiological stress response systems [15, 16], taking a toll on the body and leading to increased risk of disease and mortality [17]. An established biological index of chronic stress exposure is allostatic load (AL) [15]. AL has been linked to a higher risk of mortality, cardiovascular disease, and both physical and cognitive decline and is thus an important mechanism through which psychosocial experiences become embedded under the skin to affect health [16].

Research has also demonstrated racial differences in AL, indicating that Black Americans and other racial minorities have higher AL and faster accumulation of AL throughout their life course [18, 19]. While, to date, few studies have examined racial climate stress and AL, past studies have found positive associations between the related phenomenon of institutional discrimination and AL. In a nationally representative sample of twin pairs, perception of institutional racial inequality was related to higher AL level even after controlling for various correlates of poor health such as smoking, health behavior, and socioeconomic status [20]. Similarly, higher levels of perceived institutional discrimination by police and courts in Puerto Rican adults living in the continental U.S. were related to higher levels of AL [21].

### Institutional Belonging as a Moderator

At the same time, the relation between racial climate stress and health is not likely to be universal, and may change depending on one’s investment in and relationship with the institution. Of particular interest, institutional belonging, defined as an individual’s sense of affiliation, connection, and membership towards a group or institution [22] is likely to shape the impact of racial climate stress. Because positive group membership informs an individual’s self-esteem and self-concept [23], feelings of belonging to a group identity have been shown to protect against negative effects of a threat to the individual such as discrimination or a negative racial climate [24]. Indeed, feelings of belonging have often been conceptualized as a buffer against deleterious effects of discrimination [1], protecting against various mental and physical concomitants of discrimination [7].

However, recent research has shown that feelings of institutional belonging can be detrimental when individuals experience racial climate stress within the institution they belong to [2]. At high levels of belonging, experiencing discrimination from the group that one identifies with can cause uncertainty about one’s self and group-concept [24, 25]. This uncertainty can lead to feelings of stress and anxiety [26], ostracism [27, 28] and threats to social connections [29]. For example, individuals who felt a strong sense of belonging to their college but also faced institutional discrimination demonstrated increased cardiometabolic risk [2]. On the other hand, when one does not identify with, or feel a sense of belonging to a specific group, threats from that group may not be as detrimental to the self [24, 25]. This body of work suggests that belonging during college, a critical stage of identity development [30, 31], may interact with racial climate stress to predict health at different levels of belonging. Higher levels of belonging may exacerbate relations between racial climate stress and health, consistent with past research on institutional discrimination and health (i.e., [20, 21]). On the other hand, those with low levels of belonging may be less affected by institutional related stress.

**Fig. 1:**
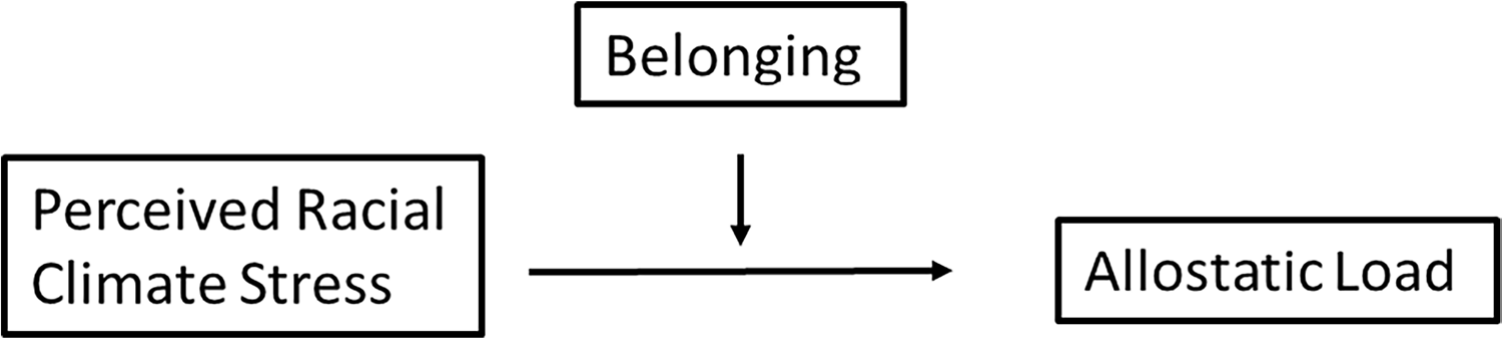
Conceptual model

In the present study, we investigated racial differences in racial climate stress, and institutional belonging in a sample of college students at a predominantly White institution, relations between perceived racial climate stress and chronic physiological dysregulation (allostatic load), and the moderating role of belonging. We hypothesized that 1) Black students would report higher levels of racial climate stress and lower levels of institutional belonging, 2) Racial climate stress would be associated with increases in AL and that institutional belonging would moderate this relationship such that individuals who felt a strong sense of belonging would have the highest levels of AL in the context of high perceived racial climate stress (see Fig. 1 for hypothesized model). We also explored the extent to which the patterns of relations vary between Black and White students.

## Methods

### Participants and Design

Students (*N* = 235 Black students and an equally sized stratified random sample of White students) were invited to participate in the study. First-generation White college students (neither parent graduated from college) were over-sampled to ensure that race and parent education would not be confounded. Of the 235 invitations sent to Black students, 68 Black students participated in the study, resulting in a 29% response rate, and 82 White students from the stratified random sample participated, resulting in a 35% response rate.

The present study consisted of 150 college freshmen and sophomores (56% female; *Mage =* 18.77 *SDage =* .80; 54.7% White; 45.3% Black), who participated in the College Student Health Study [32, 33]. Data were collected from a predominantly White Midwestern university in the USA (3% Black, 4% Asian, 4% Hispanic/Latino, 1% Native American). Socioeconomically, participants mirrored the income levels of college students from the institution with a median parental income of $90,000 to $94,999 per year (see Table 1 for breakdown of demographic and key study variables by race).

**Table 1:** Study sample characteristics

	% (*n*)	*M*	SD
Gender (female)			
Black	52.44% (41)		
White	60.29% (43)		
Age			
Black		18.85	0.87
White		18.71	0.75
Racial climate stress			
Black		8.79	5.84
White		2.15	2.72
Belonging			
Black		5.18	0.97
White		5.42	1.00
Allostatic load			
Black		1.22	1.18
White		0.94	0.93
Income		Median	
Black		$65,000–$74,999	
White		$110,000–$119,000	

### Procedure

Before participating in the study, informed consent was obtained from all participants. Various measures of participant health were then collected by research assistants and nursing staff in the clinical research unit of an on-campus university hospital in order to compute AL (please see measures section for details of AL measure). First, blood samples and anthropometric measurements were collected. After a minimum of 45-min post-blood draw, participants completed an autonomic nervous system (ANS) functioning assessment (see [34]). Participants then responded to a final questionnaire where they were asked to report on stress experienced from the racial social climate of their educational institution. After the visit, participants were given insulated containers with cold packs to collect overnight urine samples in their homes. Upon returning the urine samples, participants were debriefed and compensated 75 dollars for their participation. The research protocol was authorized by the University's institutional review board.

## Measures

### Allostatic Load

An index of allostatic load (AL) was used to assess the cumulative physiologic effect of chronic stress exposure. The index consisted of six measures: 12-h overnight urinary epinephrine and norepinephrine (indicators of ANS functioning), indicators of sympathetic adrenal medullary (SAM) axis activation; 12 h, overnight urinary cortisol, an indicator of hypothalamic-pituitary-adrenal (HPA) axis activation; body mass index (BMI), and resting systolic and diastolic blood pressure (BP), indicators of cardiometabolic risk.

University of Wisconsin Health Laboratory Services conducted initial sample processing or the urine samples. The samples were then sent to ARUP Laboratories to complete assay procedures following established protocols. Samples were first acidified. Assays were then conducted using quantitative high-performance liquid chromatography/tandem mass spectrometry. Intra-assay and interassay coefficients of variation for samples are 6.4% and 7.1% for epinephrine and 6.2% and 6.8% for norepinephrine. In addition, norepinephrine and epinephrine values were adjusted for creatinine concentrations and measured in micrograms of catecholamine/gram of creatinine (μg/g).” Cortisol was assessed using a radioimmunoassay. BP was assayed with computerized readings (Dinamap Model Pro 100, Critikon) taken at two-minute intervals while participants sat at rest. As is consistent with methodology from field studies collecting allostatic load in young adults [35], seven readings were collected, with the first reading not used, which has been shown to yield a highly reliable index of chronic resting blood pressure levels [36]. The second to seventh readings were averaged and utilized as the index for resting blood pressure. Lastly, BMI (kg/m^2^) was determined through measures of weight and height taken by training nursing staff.

In accordance with previous literature [19, 37–39] AL was calculated as the number of biomarkers in which participants scored higher than the 75th percentile in the sample. The possible range in AL scores was 0 to 6.

### Sense of Belonging

Sense of belonging at college was assessed using six items compiled and modified from three different measures: belonging uncertainty and social fit scale [40, 41], campus climate questionnaire [42], and initial college experience questionnaire [43]. Items were as following: (1) When something bad happens, I feel that maybe I don’t belong at this college (reverse scored); (2) I fit in well at this college; (3) I like the social environment at this college; (4) I am uncomfortable about being different from others at this college (reverse scored); (5) I am pleased about my decision to attend this college; (6) I feel like I belong in the university community. Responses were on a seven-point scale from “strongly disagree” to “strongly agree.” A mean of the six items was taken with higher scores indicating greater sense of belonging at college. Internal consistency was good (Cronbach’s alpha = .77) and item analysis suggested that a one factor solution fit the data adequately (report fit stats of one factor confirmatory analysis).

### Perceived Racial Climate Stress in College

Perceived racial climate stress was assessed by combining a six-item measure of *racial tension* from the Cultural Attitudes and Climate Questionnaire [8], and a six-item measure of *campus social climate* from the Minority Status Stress Scale [44]. The two measures are established indicators of *racial climate*. Examples of the *Racial Tension* items include “interracial tensions in the classroom,” and “racial conflict on campus.” Examples of the *Campus social climate* items include “having very few students of my race in my classes,” and “the university lacking concern and support for the needs of students of my race.” The original agree-disagree response format for the *racial tension* scale was modified to the format of the campus social climate scale due to our focus on assessing the perceived stressfulness of the campus environment. For all items, participants therefore indicated on a six-point scale ranging from “does not apply” (coded as 0) to “extremely stressful” (coded as 5). Item scores were summed to form an overall composite score for racial climate stress. Internal consistency for the combined measure was excellent (Cronbach’s alpha = .90).

### Covariates

Age, sex, race, and parental income were self-reported, with race verified through the university’s records. Parental income was reported on a 28-category scale with brackets of $5,000 ranging from less than $5,000 to over $200,000.

## Results

### Data Preparation

Missing data were identified for 19 participants. Mice plots were generated, revealing no discernable patterns to the missing data. An independent samples t-test (2-tailed) revealed that participants with missing data (*M* = 4.69; SD = 5.07) reported significantly less racial climate stress than participants without missing data (*M* = 9.71; SD = 9.09) *t* [136] = −2.44, *p* = .016, *d* = −0.95. There were no other differences between participants with some missing data and those with complete data. To address missing data, Full Information Maximum Likelihood (FIML) analyses were conducted using the lavaan package in R [45]. Traditional regression analyses with listwise deletion of cases with missing data yielded comparable results. Regression assumptions of homoscedasticity and lack of multicollinearity were found to be met. Univariate and multivariate normality of data was checked and corrected for. Values three standard deviations away from the mean in age and income were winsorized and income was then log-transformed. After transformation, analyses of all variables concluded that skew and kurtosis were well within normal limits and therefore analysis proceeded.

### Group Differences and Covariate Identification

In investigating main group differences, independent samples t-tests (2-tailed) revealed that Black participants (*M =* 8.79; SD = 5.84) reported significantly higher levels of racial climate stress than White participants (*M =* 2.15, SD = 2.71), *t* [136] = −8.94, *p* < .001, *d* = −1.54, that Black participants (*M =* 2.64; SD = .54), but did not show significantly different levels of institutional belonging (*M =* 5.67, SD = 1.00) compared to White participants (*M =* 5.18, SD = 0.97), *t* [139] = 1.46, *p* = .15, *d* = 0.25, partially supporting hypothesis 1. Additionally, Black participants reported significantly lower income compared to White participants (*M=*3.09; *SD=* .46), *t* [138] = 3.69, *p* < .001, *d* = 0.63, Further independent samples t-tests (2-tailed) demonstrated a marginally significant sex difference in AL. Biological females (*M =* 1.20; SD = 1.11) had slightly higher mean AL scores compared to biological males (*M =* .89; *SD =* .98) *t* [148] = −1.78, *p* = .077, *d* = −0.29. Additionally, correlations computed with FIML revealed that age was positively associated with racial climate stress *r*(150) = .19, *p =* .03, and that racial climate stress was negatively correlated with perceived institutional belonging *r*(150) = −.27, *p =* .003 (see Table 2 for correlations). Given these associations, sex, age, and income were included in analyses as covariates.

**Table 2:** Means, standard deviations, and correlations for continuous variables

Variable	M	SD	1	2	3	4	5
1. Racial climate stress	4.94	5.41	__				
2. Belonging	5.32	0.99	−0.27*** [−.43, −.11]	__			
3. Age	18.77	0.80	0.18* [.02, .34]	−0.02 [−.19, .14]	__		
4. Household income	2.82	0.52	−0.12 [−.30 .06]	0.15 [−.02, .31]	−0.17* [−.33, −.01]	__	
5. Allostatic load	1.07	1.06	0.20 [−.03, .44]	0.08 [−.09, .25]	−0.12 [−.27, .05]	−0.06 [−.23, .11]	__

Discrimination = racial climate stress; belonging = perceived institutional belonging. ^*^p < .05; ^**^p < .01

### Relations Between Racial Climate Stress and Allostatic Load

A regression analysis using FIML to address missing data was performed to examine the relationship between perceived racial climate stress and allostatic load, with race, sex, age, and parents’ combined income entered as covariates. This model did was not significant (*R*^*2*^ = .059, AIC = 2251.854, Model Fit Statistic (5) = 9.10, *p* = .11). Moreover, racial climate stress was not a significant predictor within this model (*β* = .010, *p* = .656, 95% CI = −0.03–0.05; see Table 3 for regression statistics). These non-significant results were comparable when tested in a traditional regression and listwise deletion of cases with missing data (*ΔR*^*2*^ = .000, *F*(1,127) = .019, *p* = .89; *β* = .015, *p* = .892). Thus, the hypothesis that racial climate stress would be associated with increases in AL was not supported.

**Table 3:** FIML multiple regression analyses of the relationship between al, social climate stress, belonging, and race

Predictor	Model 1				Model 2				Model 3			
	*β*	SE	*z*	*p*	*β*	SE	*z*	*p*	*β*	SE	*z*	*p*
Racial climate stress	0.01	.02	0.45	.656	0.04	.02	1.70	.089	.05	.05	1.17	.244
Age	−0.20	.11	−1.78	.075	−0.15	.11	−1.40	.162	−0.17	.11	−1.49	.137
Household income	−0.18	.18	−0.99	.323	−0.13	.18	−0.72	.473	−0.11	.18	−0.61	.544
Sex	0.22	.18	1.27	.206	0.21	.17	1.21	.227	0.20	.17	1.19	.234
Race	0.18	.23	0.78	.433	0.10	.22	0.45	.649	0.05	.24	0.23	.819
Belonging					0.08	.09	0.90	.366	0.19	.17	1.11	.266
Racial climate stress × Belonging					0.05	.02	2.73	.006	0.08	.04	1.87	.061
Race × Racial climate stress									−0.02	.05	−0.32	−.748
Race × Belonging									−0.12	.24	−0.48	.629
Racial climate stress × Belonging × Race									−0.04	.05	−0.82	.414

Belonging = perceived institutional belonging. Fit for model 1: *R*^*2*^ = .059, *AIC* = 2251.854*, Model fit statistic* (5) = 9.10, *p* = .105. *N* = 150. Fit for model 2: *R*^*2*^ = .11, *AIC* = 4189.74, *model fit statistic* (9) = 16.42, *p* = .06. Fit for Model 3: *R*^*2*^ = .12, *AIC* = 4753.34, *model fit statistic* (10) = 17.07, *p* = .07. *N* = 150

**Fig. 2:**
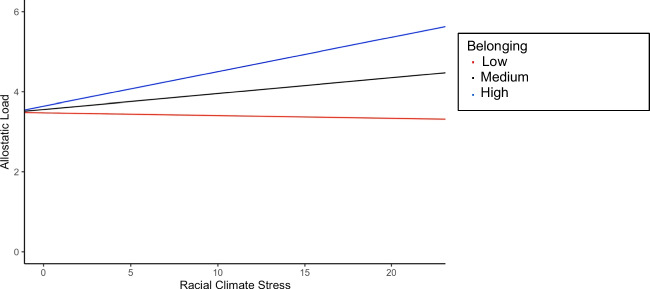
Simple slopes analysis of the relationship between al and racial climate stress at varying levels of belonging

To examine the moderating role of institutional belonging in the relationship between racial climate stress and allostatic load (hypothesis 2), another regression analysis using FIML was conducted. Institutional belonging was entered as the moderator, by computing the product of the racial climate stress variable, and belonging after centering, with race, sex, age, and parents’ combined income as covariates. The interaction term was significant, where results demonstrated that institutional belonging significantly moderated the relationship between racial climate stress and AL (*β*_*int*_ = .05, *p* = .006, 95% CI = 0.01 – 0.09). Simple slopes analyses found that the positive relationship between racial climate stress and AL was significant for individuals who reported a strong sense of belonging (*β* = .09, *p* = .011), whereas the relationship was not significant for those who had moderate (*β* = .04, *p* = .089) or low levels of belonging (*β* = −.01, *p* = .763, supporting hypothesis 2 (see Fig. 2 for interaction visualization, and Model 2 in Table 3 for estimates).

A post-hoc power analysis was conducted to ensure there was significant power to detect a two-way interaction with *N* = 150 subjects, an observed effect size of *R*^2^ = .11, and seven predictors including covariates. The analysis indicated observed power was .89, indicating there was sufficient power to detect an effect.

The moderating effect of race was also investigated in this analysis. Race did not prove to significantly moderate the relationship between institutional belonging and AL (*β*_*int*_ = -.12, *p* = .629, 95% CI = -0.52 – 0.40), or racial climate stress and AL (*β*_*int*_ = −.02, *p* = .748, 95% CI = −0.10 – 0.10), suggesting that these patterns did not vary across race. Comparable results were found using the PROCESS 3.5 macro for SPSS, model 1 with listwise deletion of cases with missing data ([46]; *R*^*2*^ = .11, *F*(7, 123) = 2.16, *p* = .043; *β*_*int*_ = .05, *p* = .006).

Finally, to explore the relations between the three-way interaction between race, institutional belonging, and racial climate stress and AL, another regression analysis using FIML was conducted. All previous IVs, moderators, interaction terms, and covariates were entered into the regression model, with the addition of the product of the dummy coded race variable, belonging, and racial climate stress variables. Results demonstrated that the three-way interaction was not significant (*β*_*int*_ = −.04, *p* = .414, 95% CI = −0.14 – 0.06) (See Table 3). We also explored quadratic relations testing whether both low and high levels of belonging may exacerbate the effects of perceived racial climate stress on AL. None of these models were significant. Given research demonstrating the large sample size necessary to test a three-way interaction [47], these null results may be explained by a lack of power.

## Discussion

The present study investigated the relations between racial climate stress and allostatic load among emerging adults in the college context, and the moderating effect of feelings of institutional belonging. We hypothesized that (1) Black students would report higher levels of racial climate stress and lower levels of institutional belonging and (2) that there would a positive relationship between racial climate stress with AL, and that this relationship would be strongest for those who felt they belonged to their institution. Our data provided support for higher levels of racial climate stress among Black students and a significant interaction between discrimination and belonging for both Black and White students. For those low or medium in belonging, racial climate stress had little to no relationship with allostatic load; however, those high in belonging demonstrated a significant positive relationship between the two variables.

These patterns of results did not appear to vary between Black and White students. Although Black students did report significantly higher levels of racial climate stress than White students, we did not find support for a significant interaction effect between race and belonging and race and racial climate stress on health, or a significant three-way interaction in our exploratory analysis of the relationships between race, racial climate stress, belonging, and health. These non-significant results indicate that perceiving a negative social climate interacts with belonging in a similar way for White and Black students.

These results also contribute to the growing literature surrounding the relationship between racial climate stress and health. Although a robust literature documents associations between individual discrimination and poor physical health [48–50], including at the physiological level [51], research is generally much sparser concerning institutional level variables such as racial climate stress [21, 52]. Understanding structural level variables is important as it informs change at the institutional level, rather than placing the burden on individuals (e.g. [53]). Our data further support the idea that belonging, while generally conceptualized as protective, can serve as a vulnerability factor consistent with social psychological theories and work by Hussain et al. [2] suggesting that there are physical health costs to increased belonging in certain contexts.

Social psychological research provides background for understanding the detrimental role of belonging. Identity centrality, or salience and importance of a group identity [54], has been shown as key to experiencing effects associated with identity threats. For example, if an identity an individual belongs to is being threatened (i.e., through racial climate stress), but is not particularly central to an individual, there will be fewer negative consequences for the self-concept [55]. On the other hand, if an identity is very central, minor threats will cause intense uncertainty and stress. Identity centrality may therefore explain why those who felt strongly about their belonging to their institution had strong negative effects related to racial climate stress. Therefore, future research should investigate identity centrality as a boundary condition for these effects on physical health.

At the same time, our results showed no significant main relationship between racial climate stress and AL, which is inconsistent with past literature demonstrating associations between racial climate stress and health, (i.e., AL, [21]; breast cancer incidence, [52]). Past literature (e.g., [52]) has utilized a life course perspective on racial climate stress asking participants to rate how frequently they have experienced racial climate stress throughout their lifetime. In contrast, our study was limited by a narrower period, asking participants only to report their perceptions of racial climate stress within the past semester. The study was also limited in its sample, only including Black and White students and first and second-year students, reducing generalizability. Given that allostatic load represents the cumulative effect of chronic stress, it is possible that stressors experienced within the previous few months or recent semesters in school for new students would not have enough time to significantly influence AL, which limited our ability to detect a main effect of racial climate stress on AL. Therefore, future studies could investigate the relationship between racial climate stress and AL over a longer time span from when students first enter college to when they graduate, to test cumulative effects on AL.

It is also important to note that our sample was collected in one specific university context, students may have self-selected into the study, limiting generalizability. Despite these limits to the scope of the sample, the response rate was larger than expected for a mail/email list recruitment strategy (e.g., see Kaplowitz et al., [56]), and the sample was deemed to be representative of the wider college population. Although there is a possibility of response bias as response rates were less than 100%, given the sample characteristics and sampling procedure, we expect the results of the study to be generalizable to college campuses with similar racial breakdowns.

Our study also found no interactive effects of race in the relationship between perceived social climate stress, belonging, and AL, demonstrating that the pattern of results do not appear race specific. These non-significant results support research showing that White students can also experience stress from the institutional climate [14, 57]. Although this research has been limited to investigating educational outcomes rather than health outcomes, our results provide support for their theory in a health context. As recent research has almost exclusively focused on the link between racial climate stress and health for minority individuals, future studies could begin to investigate the mechanisms behind adverse effects for White as compared to ethnic-racial minority individuals to elucidate further these relationships.

Despite the strengths of the current study (e.g., use of an objective marker of physiological functioning, testing unexplored relationships) we are limited in its correlational design, and thus unable to draw causal conclusions. Manipulating exposure to racial climate stress, or belonging, perhaps in a laboratory context can increase assurances for causality. In addition, our studies were underpowered for an examination of three-way interactions due to limited sample size, indicating a potential for Type II error [47]; therefore, increasing sample size by recruiting both Black and White students could potentially uncover significant effects and shed further light on the relationships between these constructs. Relatedly, given the design of our study, we were not able to test complex models investigating potential mechanisms or alternative models. For example, conceptually, belonging could also serve as a mediator between racial climate stress and AL, future longitudinal studies with larger sample sizes should test these alternative theories. Finally, we are limited in our conclusions in that our data were collected at a predominantly White institution in the Midwest. Given that perceptions of racial climate stress are likely to vary across geographical locations, as well as across institutions (i.e., predominately White vs. historically Black colleges and universities), we cannot generalize our findings to all contexts. Future research examining how these patterns may be affected by types of institutions and geographical location would improve generalizability of our inferences.

Future research could expand our understanding of the relationships between racial climate stress and belonging for other racial groups and contexts. Previous studies have investigated racial climate stress experienced in the context of police interactions, jobs, and housing (e.g., [58]) rather than racial climate stress from colleges. Facing marginalization from these larger societal institutions may have a greater and longer-term, effect on chronic stress and socioeconomic status compared to college discrimination, and likely exact a larger toll on AL. Thus, expanding our investigation of these ideas in a college context by investigating outcomes for other minority individuals can help to improve our understanding of how belonging may mitigate or exacerbate the relations between racial climate stress on health. Finally, our models focused on linear relations, at the same time, it is conceptually plausible that quadratic relations and their interactions may exist. For example, both low and high levels of belonging may exacerbate the relations between perceived racial climate and AL. While, we did not find support for these models in our exploratory analyses, given the limits of our sample size, it is imperative that future research consider non-linear relations among these variables. In sum, our research highlights the importance of considering the nuances of so-called protective factors, in context, and suggests novel directions for further study in order to understand processes of stress and health.

## Data Availability

Data and material available at - https://datadryad.org/stash/share/nYZXwk_191vz3aqgduuJBL50fgBT1Af9gFIc7J0yd0E.

## Abbreviations

AL: Allostatic load
HPA: Hypothalamic-pituitary-adrenal
SAM: Sympathomedullary

## Code Availability

Code available at - https://datadryad.org/stash/share/nYZXwk_191vz3aqgduuJBL50fgBT1Af9gFIc7J0yd0E.
